# Supplementary material for: A common human MLKL polymorphism confers resistance to negative regulation by phosphorylation
Source: Nat Commun. 2023 Sep 28;14:6046. doi: 10.1038/s41467-023-41724-6 (PMC10539340; doi:10.1038/s41467-023-41724-6)
Supplement: Supplementary file 1 — Supplementary Information [file 41467_2023_41724_MOESM1_ESM.pdf]

A common human *MLKL* polymorphism confers resistance to negative regulation by phosphorylation

Sarah E. Garnish[1 2] , Katherine R. Martin[1 2] , Maria Kauppi[1 2] , Victoria E. Jackson[1 2], Rebecca Ambrose[3 4] , Vik Ven Eng[3 5], Shene Chiou[1 2], Yanxiang Meng[1 2], Daniel Frank[1], Emma C. Tovey Crutchfield[1 6], Komal M. Patel[1], Annette V. Jacobsen[1 2], Georgia K. Atkin-Smith[1 2], Ladina Di Rago[1 2], Marcel Doerflinger[1 2], Christopher R. Horne[1 2], Cathrine Hall[1], Samuel N. Young[1], Matthew Cook[7 12], Vicki Athanasopoulos[8], Carola G. Vinuesa[7 8 13-15], Kate E. Lawlor[3 4], Ian P. Wicks[1 2], Gregor Ebert[9] Ashley P. Ng[1 2 10], Charlotte A. Slade[1 2 11], Jaclyn S. Pearson[3 4 5], Andre L. Samson[1 2], John Silke[1 2], James M. Murphy[1 2] and Joanne M. Hildebrand[1 2 *]

1. The Walter and Eliza Hall Institute, Parkville, VIC, Australia
2. University of Melbourne, Department of Medical Biology, Parkville, VIC, Australia
3. Centre for Innate Immunity and Infectious Diseases, Hudson Institute of Medical Research, Clayton, VIC, Australia
4. Department of Molecular and Translational Science, Monash University, Clayton, VIC, Australia
5. Department of Microbiology, Monash University, Clayton, VIC, Australia
6. University of Melbourne, Faculty of Medicine, Dentistry and Health Sciences, Parkville, VIC, Australia
7. Centre for Personalised Immunology and Canberra Clinical Genomics, Australian National University, Canberra, ACT, Australia
8. Department of Immunology and Infection, John Curtin School of Medical Research, Australian National University, ACT, Australia
9. Institute of Virology, Technical University of Munich/Helmholtz Munich, Munich, Germany

10. Clinical Haematology Department, The Royal Melbourne Hospital and Peter MacCallum Cancer Centre, Parkville, VIC, Australia
11. Department of Clinical Immunology & Allergy, Royal Melbourne Hospital, Parkville, VIC, Australia
12. Present address, Cambridge Institute for Therapeutic Immunology and Infectious Disease, University of Cambridge, Cambridge, UK
13. Present address, The Francis Crick Institute, London, UK
14. Present address, University College London, London, UK
15. Present address, China Australia Centre for Personalized Immunology (CACPI), Renji Hospital, Shanghai Jiao Tong University School of Medicine (SJTUSM), Shanghai, China

* To whom correspondence may be addressed- jhildebrand@wehi.edu.au

Supplementary Figure 1

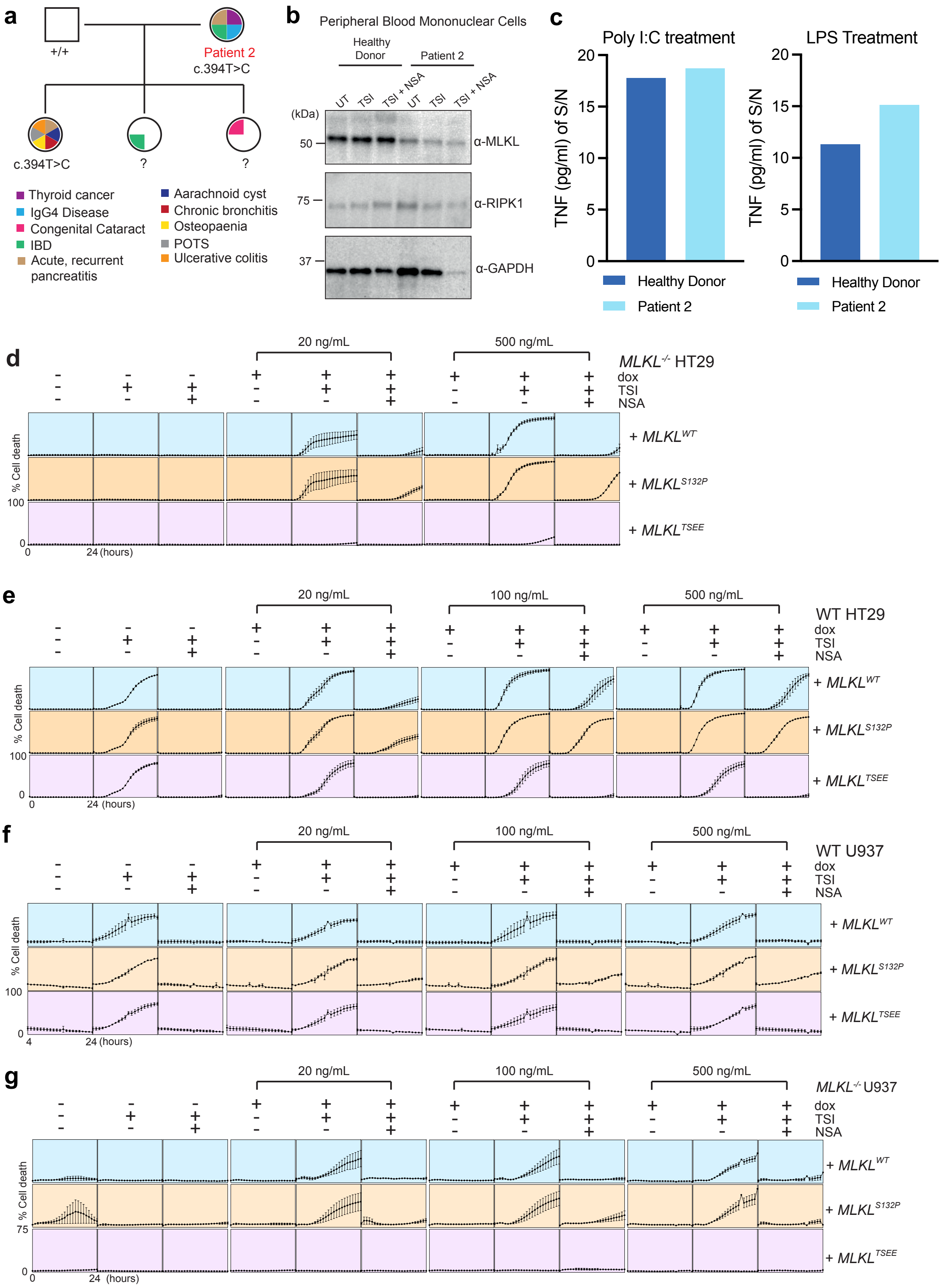

Supplementary Figure 1 (cont)

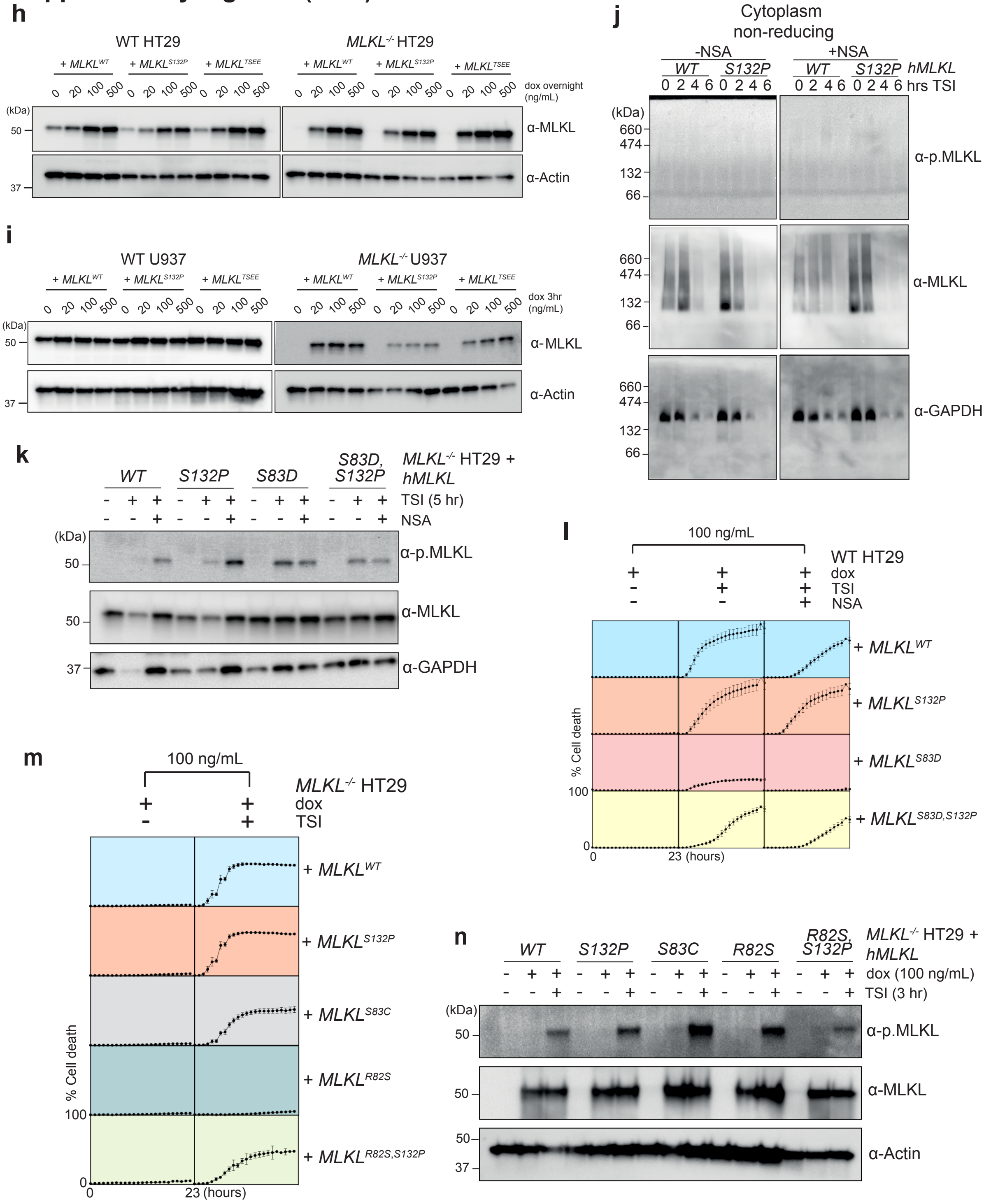

Supplementary Figure 1. MLKL^{S132P} is less sensitive to inhibition by necrosulfonamide. (**A)** Family pedigree of patient 2. Known diagnoses for family members are indicated. (**B)** Peripheral blood mononuclear cells (PBMC) isolated from patient 2 and an aged matched healthy donor control were stimulated as indicated for 4 hours for western blot analysis. (**C)** ELISA measurement of supernatant TNF in PBMCs stimulated with LPS or Poly I:C for 5 hours. Mean of technical triplicates presented. **(D-G)** Evaluation of necroptotic signaling by MLKL^{WT}, MLKL^{S132P} and MLKL^{TSEE} in *MLKL*$^{-/-}$ **(D)** or WT **(E)** HT29 cells and WT **(F)** or *MLKL*$^{-/-}$ **(G)** U937 cells. Human MLKL expression was induced with doxycycline (Dox) and cells were treated with necroptotic stimulus (TNF, Smac mimetic, IDN-6556; TSI) in the presence or absence of MLKL inhibitor necrosulfonamide (NSA; 1 μM). Cell death was measured by percentage of SYTOX Green positive cells quantified using IncuCyte live cell imaging. Independent cell lines were assayed in *n=3* experiments, with errors bars indicating the mean ± SEM. **(H, I)** Western blot analyses of induced WT or *MLKL*$^{-/-}$ HT29 **(H)** or U937 **(I)** cells expressing *MLKL*^{WT}, *MLKL*^{S132P} or *MLKL*^{TSEE}. **(J)** Blue-Native PAGE crude cytoplasm fractions of *MLKL*$^{-/-}$ HT29 cells under TSI stimulation (0-6 hours) in the presence or absence of NSA. **(K)** Western blot analyses of whole cell lysates taken 5 h post stimulation from induced *MLKL*$^{-/-}$ HT29 cells expressing wild-type or mutant *MLKL* constructs. **(L, M)** Evaluation of necroptotic signaling in WT **(L)** or *MLKL*$^{-/-}$ **(M)** HT29 cells expressing wild-type or mutant *MLKL* constructs. Cell death was measured by percentage of SYTOX-green positive cells quantified using IncuCyte live cell imaging. Independent cell lines were assayed in *n=4* experiments, with errors bars indicating the mean ± SEM. **(N)** Western blot analyses of whole cell lysates taken in the presence or absence of 3 h post TSI stimulation from doxycycline induced *MLKL*$^{-/-}$ HT29 cells expressing wild-type or mutant *MLKL* constructs. Blots images in **B, H, I, J, K** & **N** are representative images of at least two independent repeat experiments. Source data are provided as Source Data file.

Supplementary Figure 2

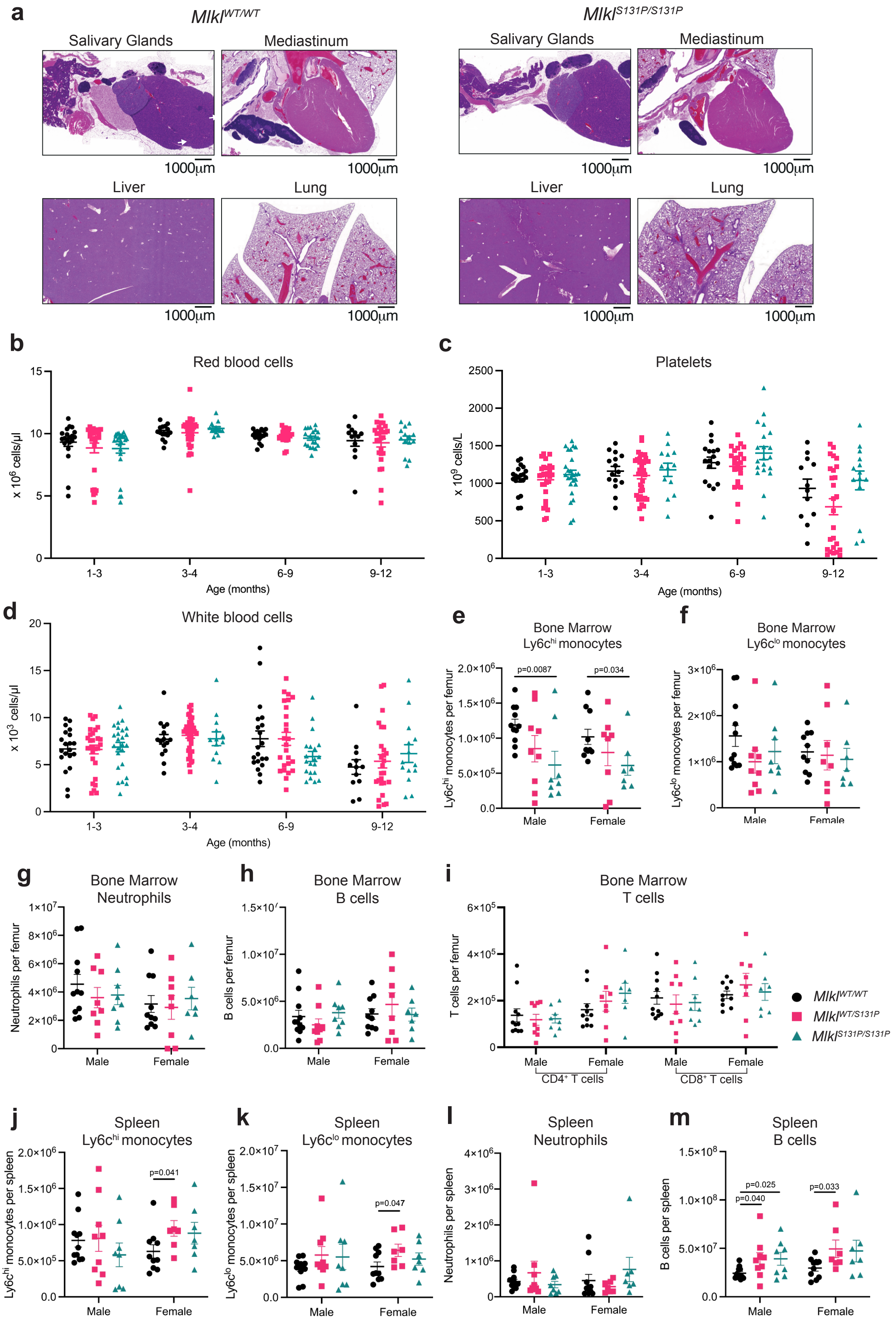

Supplementary Figure 2 (cont)

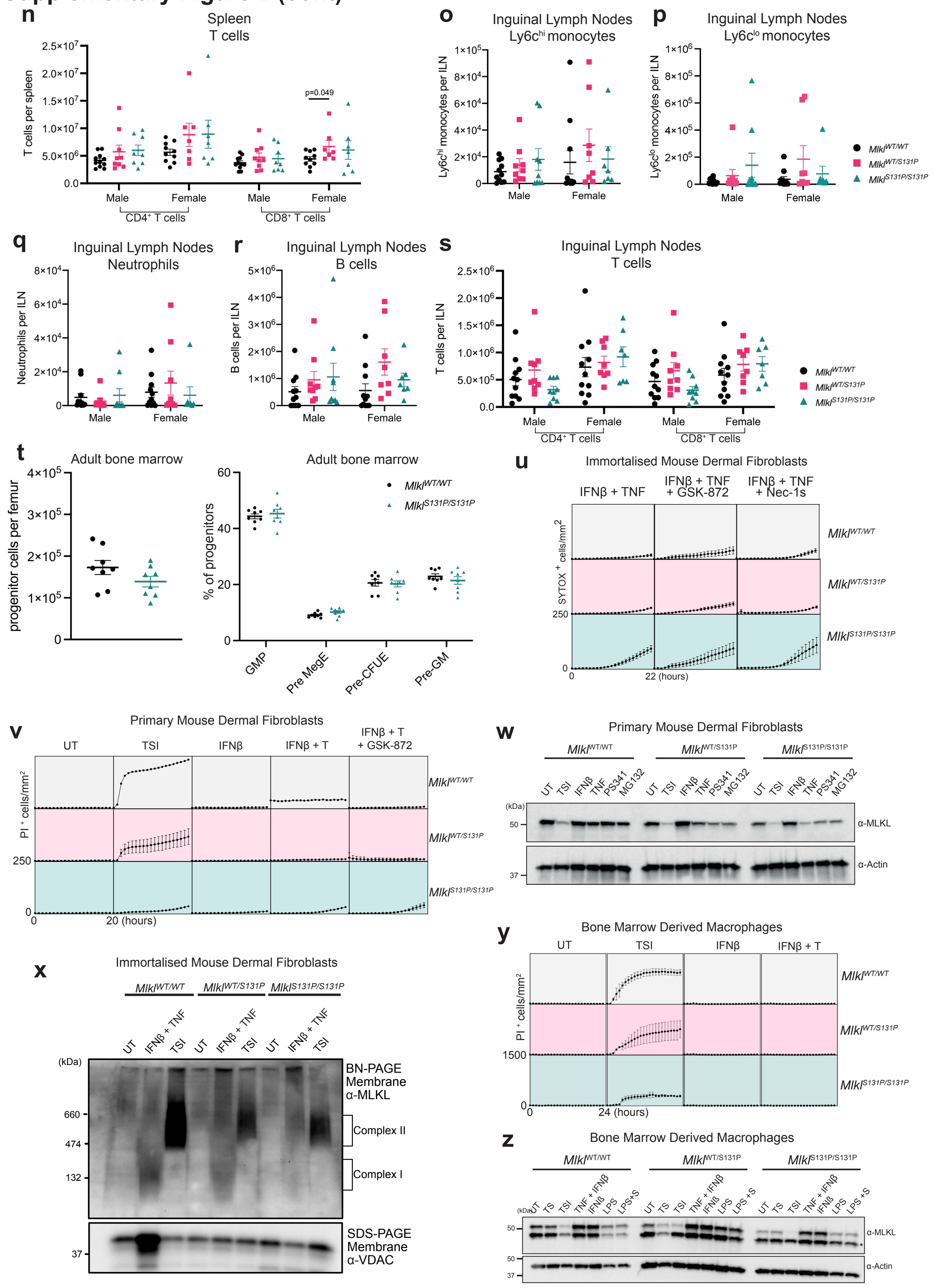

Supplementary Figure 2. Cells expressing endogenous MLKL^{S131P} exhibit reduced TSI-induced necroptotic cell death.

(A) Representative images of H&E staining of salivary glands, mediastinum, liver and lung from 7–9-month-old *Mlkl$^{WT/WT}$* and *Mlkl$^{S131P/S131P}$* mice. Images are representative of *n=2* mice per genotype. **(B-D)** ADVIA hematology quantification of circulating **(B)** red blood cells (*n=20,28,25,15,38,13,17,23,20,12,26,14*), **(C)** platelets (*n=20,29,25,15,38,13,17,23,20,12,26,14*), and **(D)** white blood cells (*n=20,28,25,15,38,13,20,25,20,12,26,14*), in *Mlkl$^{WT/WT}$*, *Mlkl$^{WT/S131P}$* and *Mlkl$^{S131P/S131P}$* mice across age. **(E-S)** Flow cytometry quantification of innate and adaptive immune cells in the **(E-I)** bone marrow (*n=11,9,8,10,8,7*), **(J-N)** spleen (*n=11,9,8,10,7,7*), and **(O-S)** inguinal lymph nodes (ILN) (*n=11,9,9,11,8,7*) of 8–12-week-old basal state *Mlkl$^{WT/WT}$, Mlkl$^{WT/S131P}$,* and *Mlkl$^{S131P/S131P}$* mice. **(T)** GMP, MegE, CFU-E, and Pre-GM progenitor populations in adult bone marrow presented as percentage of gated progenitors (Lin^{-}cKit^{+}Sca1^{-}). Data presented mean ± SEM of *n* = 8. Immortalized mouse dermal fibroblasts (MDF) were stimulated with IFNβ and TNF in the presence of RIPK3 inhibitor GSK-872 or RIPK1 inhibitor Nec-1s. SYTOX-positive cells (per mm^2) were quantified for 22 hours using IncuCyte SX5 live cell imaging **(U)**. Death data represent mean ± SEM, *n*=4 *Mlkl$^{WT/WT}$, 4 Mlkl$^{WT/S131P}$, 5 Mlkl$^{S131P/S131P}$* cell lines examined in 1, 2 or 3 independent experiments. **(V, W)** Primary MDFs were stimulated as indicated for 6 hours for western blot analysis **(V)** or 20 hours for quantification of PI-positive cells using IncuCyte S3 live cell imaging **(W)**. Death data represent mean *n*=2 *Mlkl$^{WT/WT}$, 3 Mlkl$^{WT/S131P}$, 3 Mlkl$^{S131P/S131P}$* biologically independent cell lines. Error bars for *Mlkl$^{WT/S131P}$* and *Mlkl$^{S131P/S131P}$* represent SEM. **(X)** BN-PAGE analysis of crude membrane fractions from immortalized MDFs stimulated with IFNβ + TNF (overnight) or TSI (6 hours). **(Y, Z)** Bone marrow derived macrophages were stimulated as indicated for 24 hours for quantification of PI-positive cells using IncuCyte S3 live cell imaging **(Y)** or 6 hours for

western blot analysis **(Z).** Death data represent mean ± SEM, *n=4* $Mlkl^{WT/WT}$*, 3* $Mlkl^{WT/S131P}$*, 4* $Mlkl^{S131P/S131P}$ biologically independent cell lines. (**B-T**) Each symbol represents a biologically independent mouse sampled and error bars represent mean ± SEM. *P* values are calculated using an unpaired, two-tailed Students t-test. Source data are provided as Source Data file.

Supplementary Figure 3

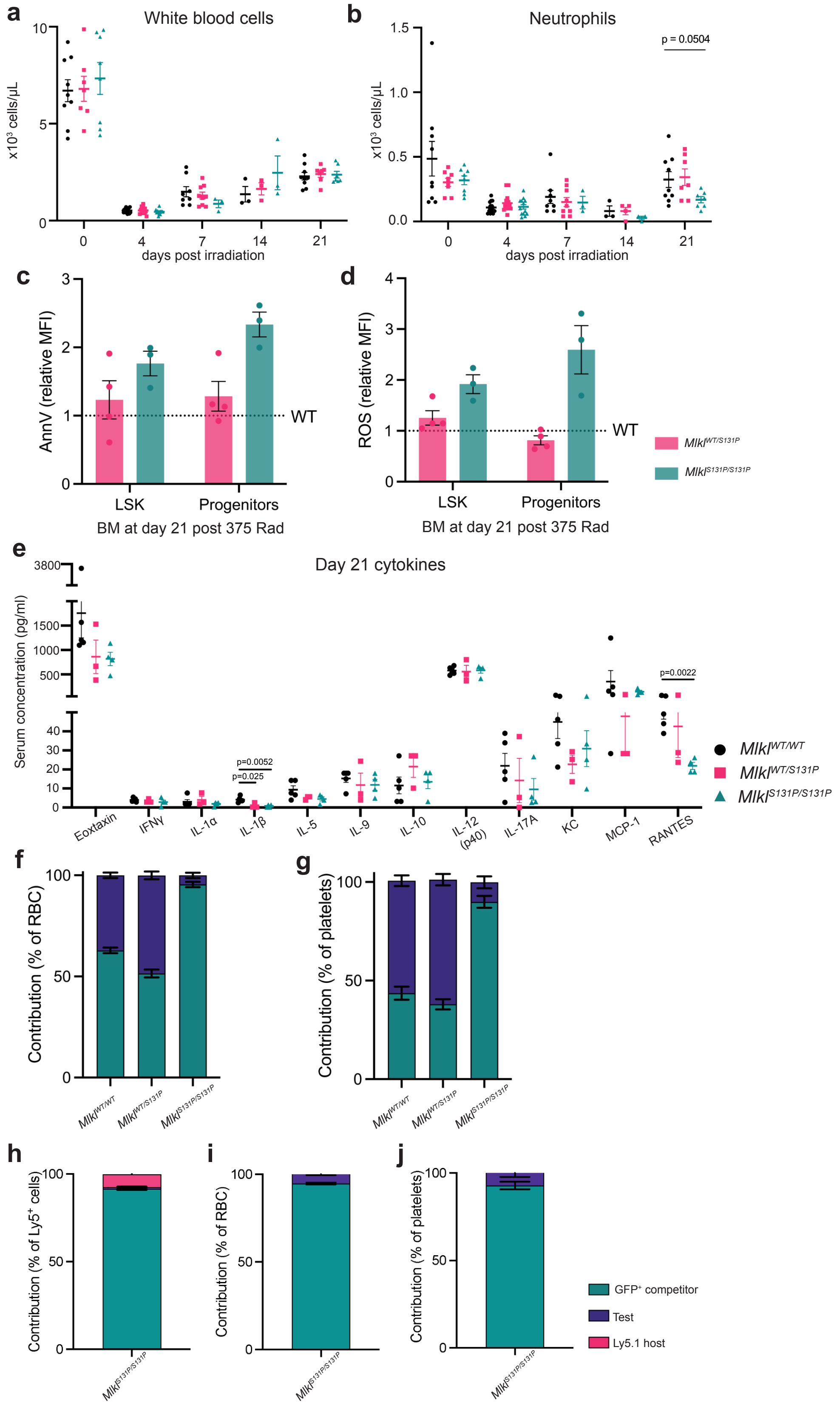

Supplementary Figure 3. ***$Mlkl^{S131P/S131P}$*** **hematopoietic stem cells transplanted in excess are outcompeted at 6-weeks post-transplant.**

(A) Peripheral white blood cells (*n=9,7,8,15,13,13,8,9,3,3,3,3,9,7,*7) and **(B)** neutrophils (*n=8,9,8,15,13,13,8,9,3,3,4,3,9,7,7*) in *$Mlkl^{WT/WT}$, $Mlkl^{WT/S131P}$,* and *$Mlkl^{S131P/S131P}$* mice following treatment with 5.5 Gy radiation. Mean ± SEM of *n*=3-15 independent mice from three separate experiments. Relative amount of Annexin V **(C)** and ROS **(D)** in *$Mlkl^{WT/S131P}$* and *$Mlkl^{S131P/S131P}$* LSK and progenitor cells was determined 21 days post irradiation. MFI calculated relative to mean of *n= 6 $Mlkl^{WT/WT}$* LSK and progenitor cells. Mean ± SEM of *n=4 $Mlkl^{WT/S131P}$* and *n=3 $Mlkl^{S131P/S131P}$* biologically independent mice. **(E)** Multiplex measurement of plasma cytokine levels at 21-days post myelosuppressive radiation. Mean ± SEM of *n=4 $Mlkl^{WT/WT}$*, *3 $Mlkl^{WT/S131P}$* and *4 $Mlkl^{WT/WT}$* biologically independent mice. Each symbol represents a biologically independent mouse sampled. Bone marrow from *$Mlkl^{S131P/S131P}$* mice on $CD45^{Ly5.2}$ background was mixed with wild-type GFP^{+} competitor bone marrow on a $CD45^{Ly5.2}$ background at a **(F, G)** 50:50 (*n=11,6,9*) or **(H-J)** 70:30 (*n=6*) ratio and transplanted into irradiated $CD45^{Ly5.1}$ recipients. Relative donor contribution to red blood cells **(F, I)**, platelets **(G, J)** and peripheral blood mononuclear cells (PBMC) **(H)** was assessed at 6 weeks post-transplantation. Mean ± SEM shown. Host contribution ($CD45^{Ly5.1}$) depicted in pink, GFP competitor in green, and test (*$Mlkl^{WT/WT}$, $Mlkl^{WT/S131P}$, $Mlkl^{S131P/S131P}$*) in purple. *P* values are calculated using an unpaired, two-tailed Students t-test. Source data are provided as Source Data file.

Supplementary Figure 4

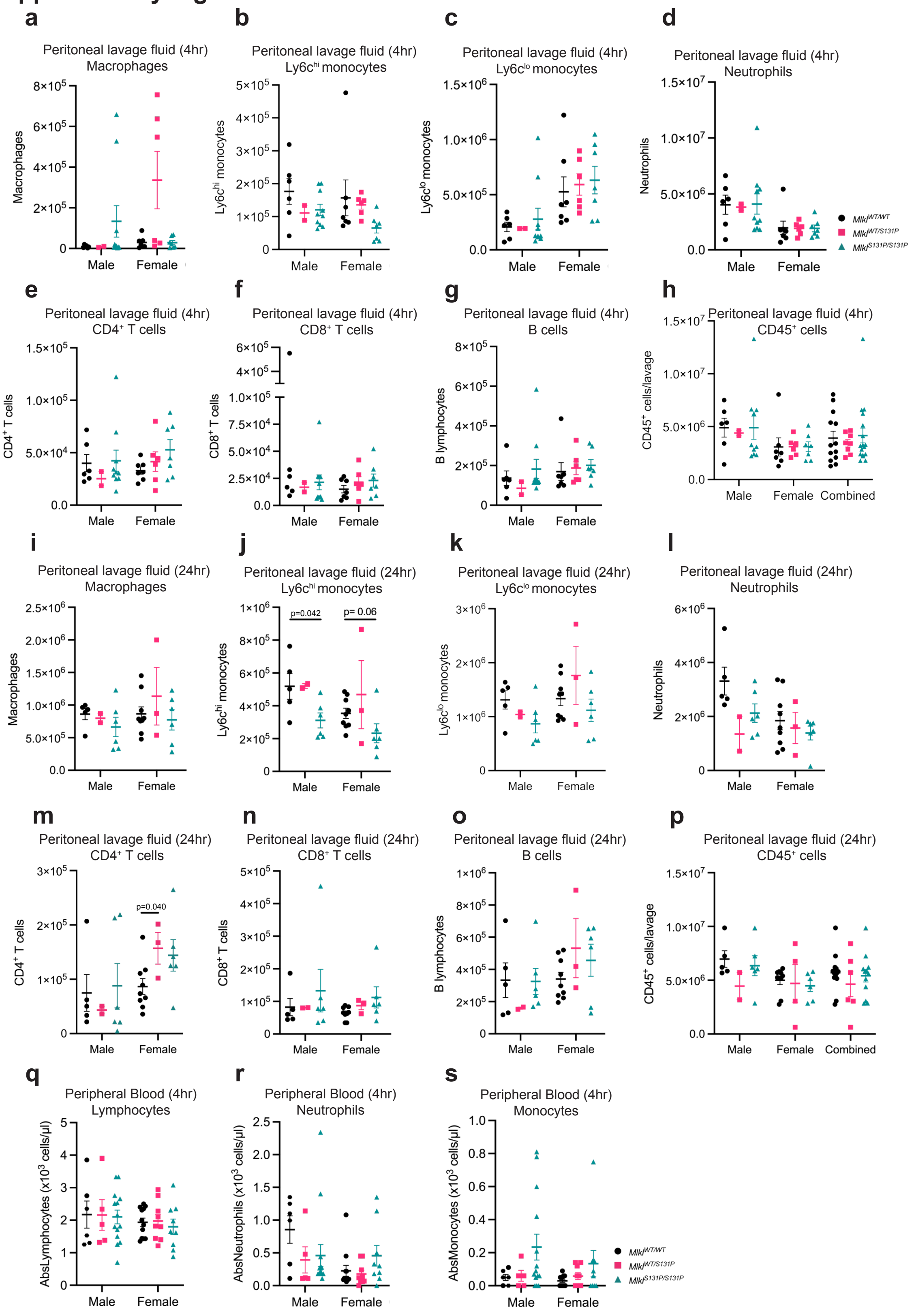

Supplementary Figure 4 (cont)

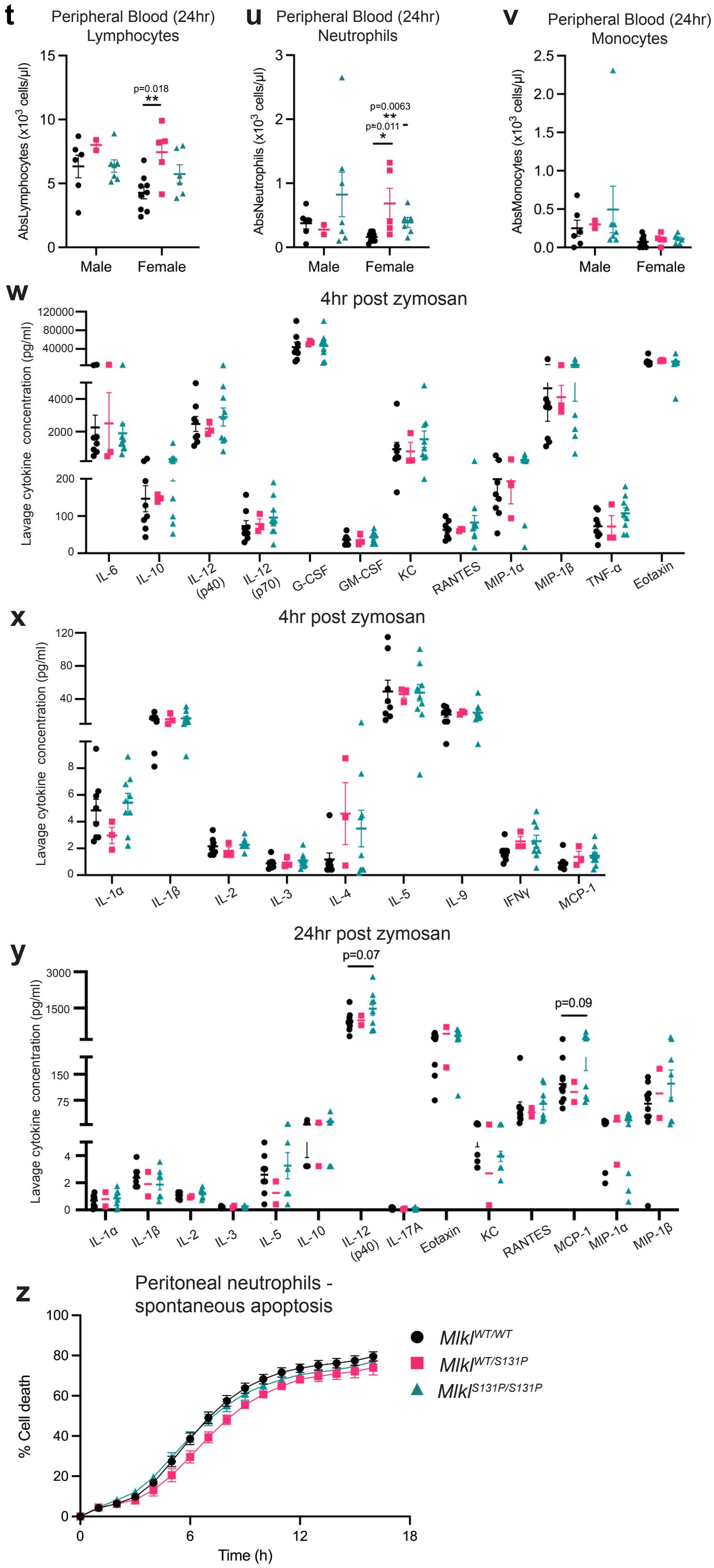

Supplementary Figure 4. ***Mlkl*^{S131P} mice have reduced levels of Ly6C^{hi} monocytes in the peritoneal lavage at 24-hours post zymosan injection.**

(A-P) Flow cytometry quantification of peritoneal innate (macrophages, Ly6C^{hi}, Ly6C^{lo} and neutrophils) and adaptive (CD4^{+} T cells, CD8^{+} T cells and B cells) immune cells at **(A-H)** 4- (*n=6,2,10,7,6,7*) or **(I-P)** 24- (*n=5,2,6,9,3,6*) hours post-intraperitoneal injection of zymosan as indicated. **(Q-V)** ADVIA hematology quantification of peripheral blood cells (lymphocytes, neutrophils, and monocytes) at **(Q-S)** 4- (*n=6,5,14,12,9,9*) and **(T-V)** 24- (*n=6,2,7,9,5,6*) post-injection of zymosan. **(W-Y)** Multiplex measurement of cytokine levels in peritoneal lavage at **(W, X)** 4- (*n=8,3,9*) or **(Y)** 24- (*n=10,2,8*) hours post-zymosan injection. Each symbol represents one biologically independent mouse, with mice from the 4- or 24-hour timepoint pooled from 3 and 2 independent zymosan experiments respectively. Error bars represent mean ± SEM. **(Z)** Evaluation of spontaneous apoptosis in neutrophils recruited and isolated from the peritoneum 4-hours post-zymosan injection. Neutrophils were left unstimulated (spontaneous apoptosis) for 16 hours and cell death was measured every hour by percentage of SYTOX Green positive cells quantified using IncuCyte SX5 live cell imaging. Data were collected from one independent experiment with male and female data pooled, neutrophils isolated from biologically independent mice with mean ± SEM of *n= 13 Mlkl*$^{WT/WT}$*, 6 Mlkl*$^{WT/S131P}$*, 14 Mlkl*$^{S131P/S131P}$. *P* values are calculated using an unpaired, two-tailed Students t-test. Source data are provided as Source Data file.

Supplementary Figure 5

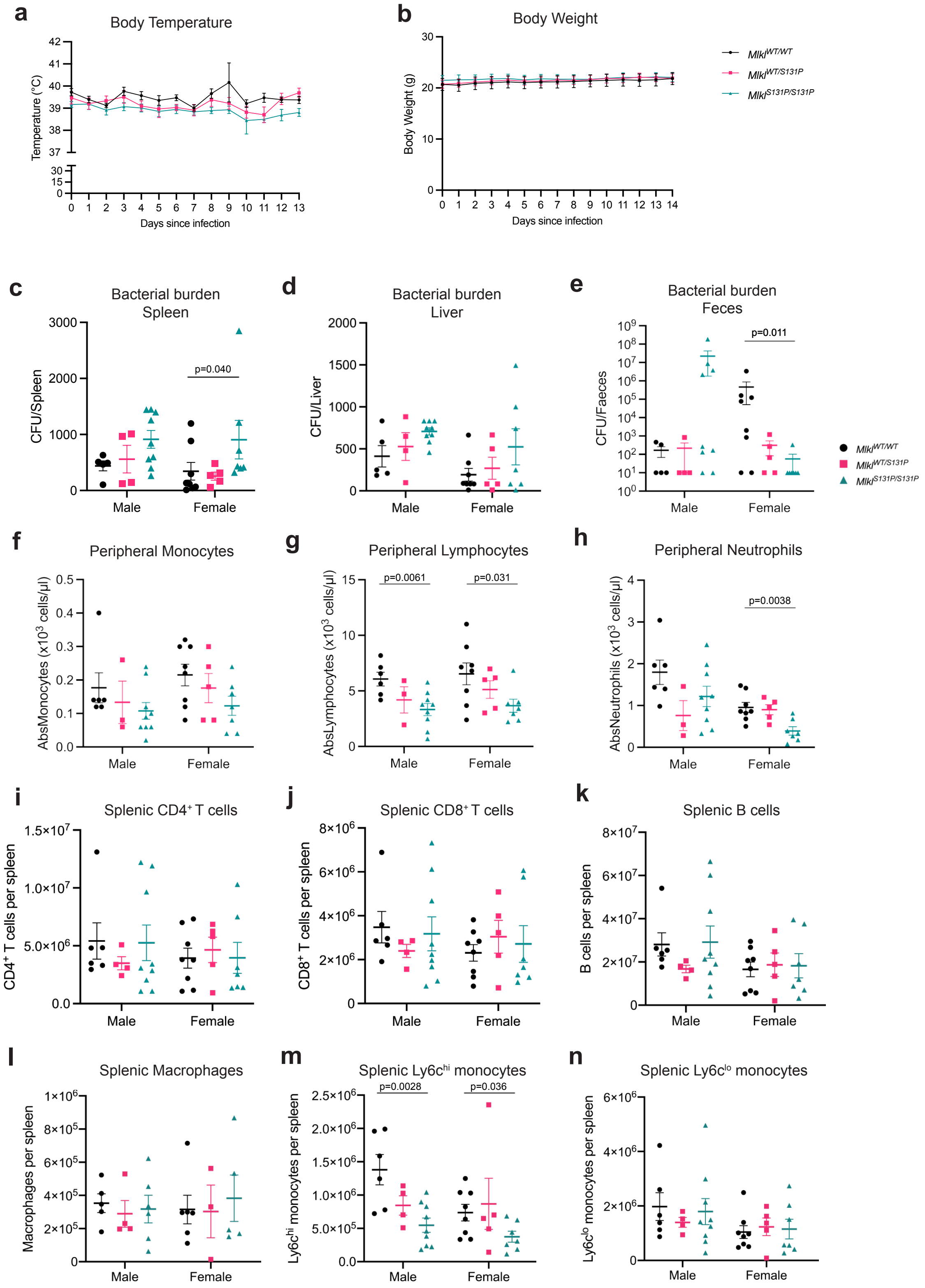

Supplementary Figure 5 (cont)

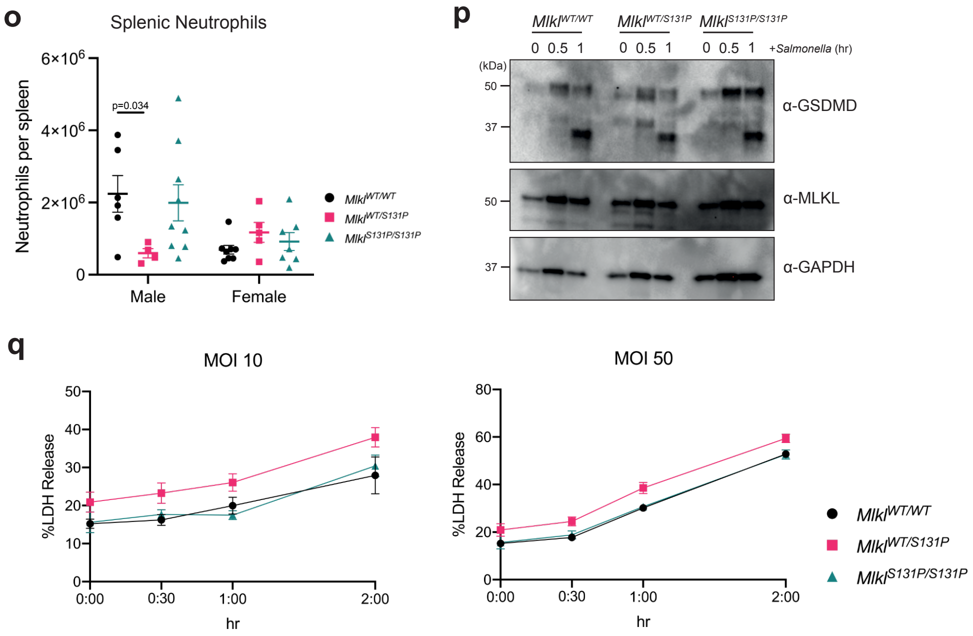

Supplementary Figure 5. ***Mlkl*** ^{S131P} **does not abrogate death of Bone Marrow Derived Macrophages upon *in vitro Salmonella* infection.**

(A, B) *Mlkl* $^{WT/WT}$, *Mlkl* $^{WT/S131P}$, and *Mlkl* $^{S131P/S131P}$ mice were infected with *Salmonella* via oral gavage and monitored by daily body temperature **(A)** and weight **(B)** measurements (*n=13,9,16*). **(C-E)** Bacterial burden calculation of *Salmonella* colonization in the spleen **(C)**, liver **(D)**, and feces **(E)** at experimental endpoint (*n=5,4,9,8,5,7*). **(F-H)** ADVIA hematology quantification of peripheral monocytes **(F)**, lymphocytes **(G)** and neutrophils **(H)** (*n=6,3,9,8,5,7*). **(I-O)** Splenic adaptive ($CD4^+$ T cells, $CD8^+$ T cells and B cells) **(I-K)** and innate (macrophages, $Ly6C^{hi}$, $Ly6C^{lo}$ and neutrophils) **(L-O)** immune cells were quantified by flow cytometry at experimental endpoint (*n=6,8,4,5,9,7*; with exception of macrophages *n=5,4,6,6,3,5*). *Salmonella* infection was completed 3 independent times, with each symbol representing a biologically independent mouse sampled. Error bars represent mean ± SEM. **(P, Q)** *In vitro* assessment of *Salmonella* SL1344 infection of primary bone marrow derived macrophages (BMDM) generated from *Mlkl* $^{WT/WT}$, *Mlkl* $^{WT/S131P}$, and *Mlkl* $^{S131P/S131P}$ mice. **(P)** BMDMs were infected with *Salmonella* (MOI:25) and cleavage associated with Gasdermin-D activation during pyroptosis was analyzed by immunoblotting at the indicated time points. **(Q)** LDH release cell death assay of BMDMs after infection with *Salmonella* (MOI:10 or MOI:50) at indicated time points. *In vitro Salmonella* experiment completed once, with mean ± SEM for *n=3* individual mice shown. Blot images in **(P)** are representative of independent duplicates. *P* values are calculated using an unpaired, two-tailed Students t-test. Source data are provided as Source Data file.

Supplementary Figure 6

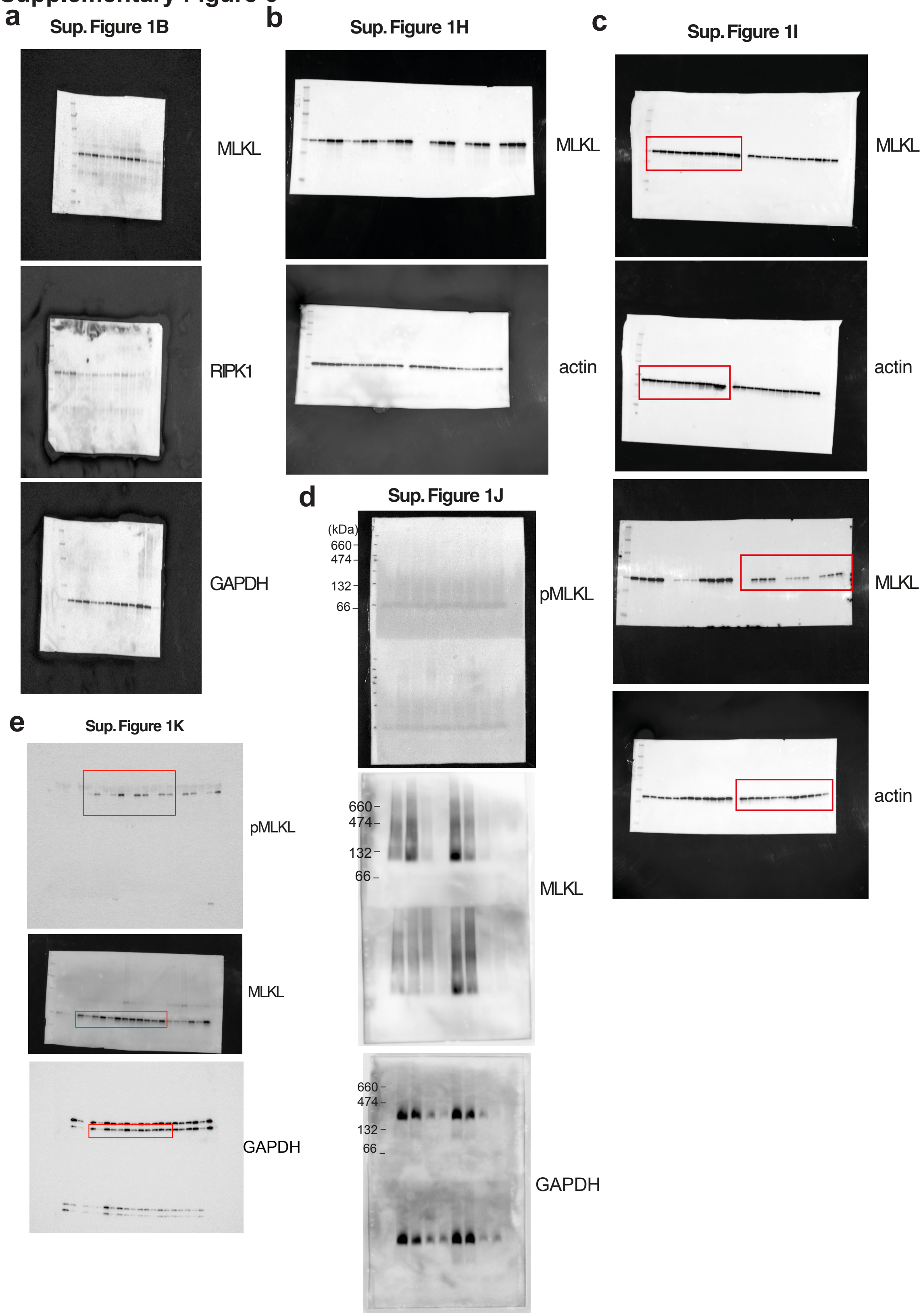

Supplementary Figure 6 (cont)

f **Sup. Figure 1N**

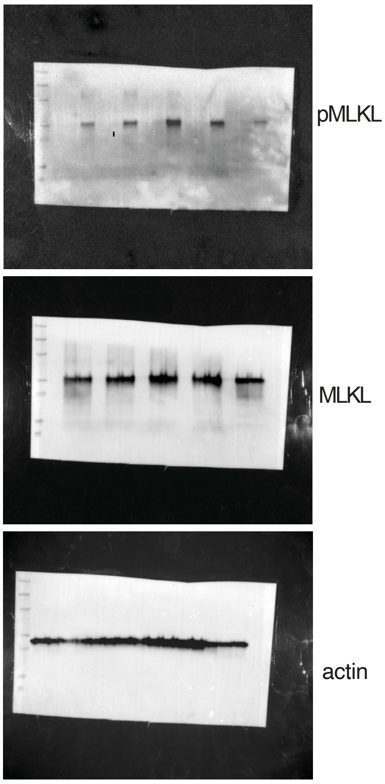

h **Sup. Figure 2X**

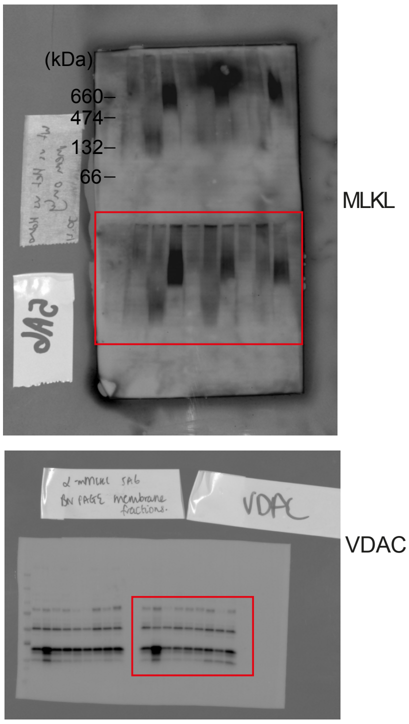

g **Sup. Figure 2W**

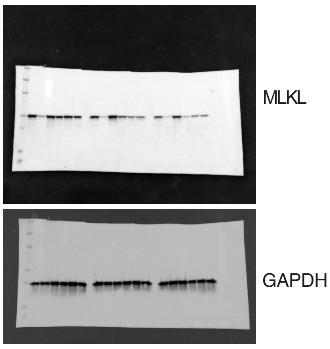

i **Sup. Figure 2Z**

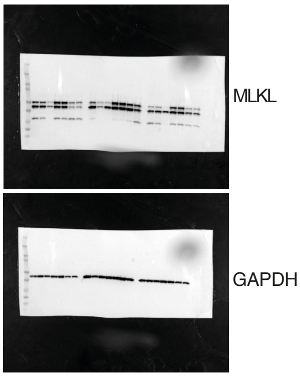

j **Sup. Figure 5P**

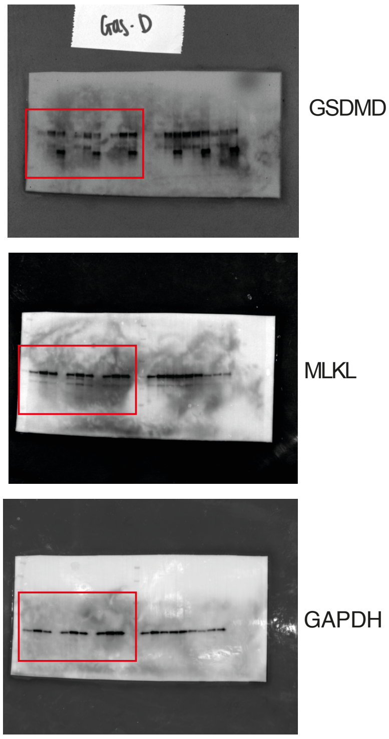

Supplementary Figure 6. Source data for gel images. The uncropped images correspond to the source data as follows. **a.** Supplementary Figure 1b. **b.** Supplementary Figure 1h. **c.** Supplementary Figure 1i. **d.** Supplementary Figure 1j. **e.** Supplementary Figure 1k. **f.** Supplementary Figure 1n. **g.** Supplementary Figure 2w. **h.** Supplementary Figure 2x. **i**. Supplementary Figure 2z. **j.** Supplementary Figure 5p. Red boxes indicate biological replicate displayed in figure.

Supplementary Figure 7

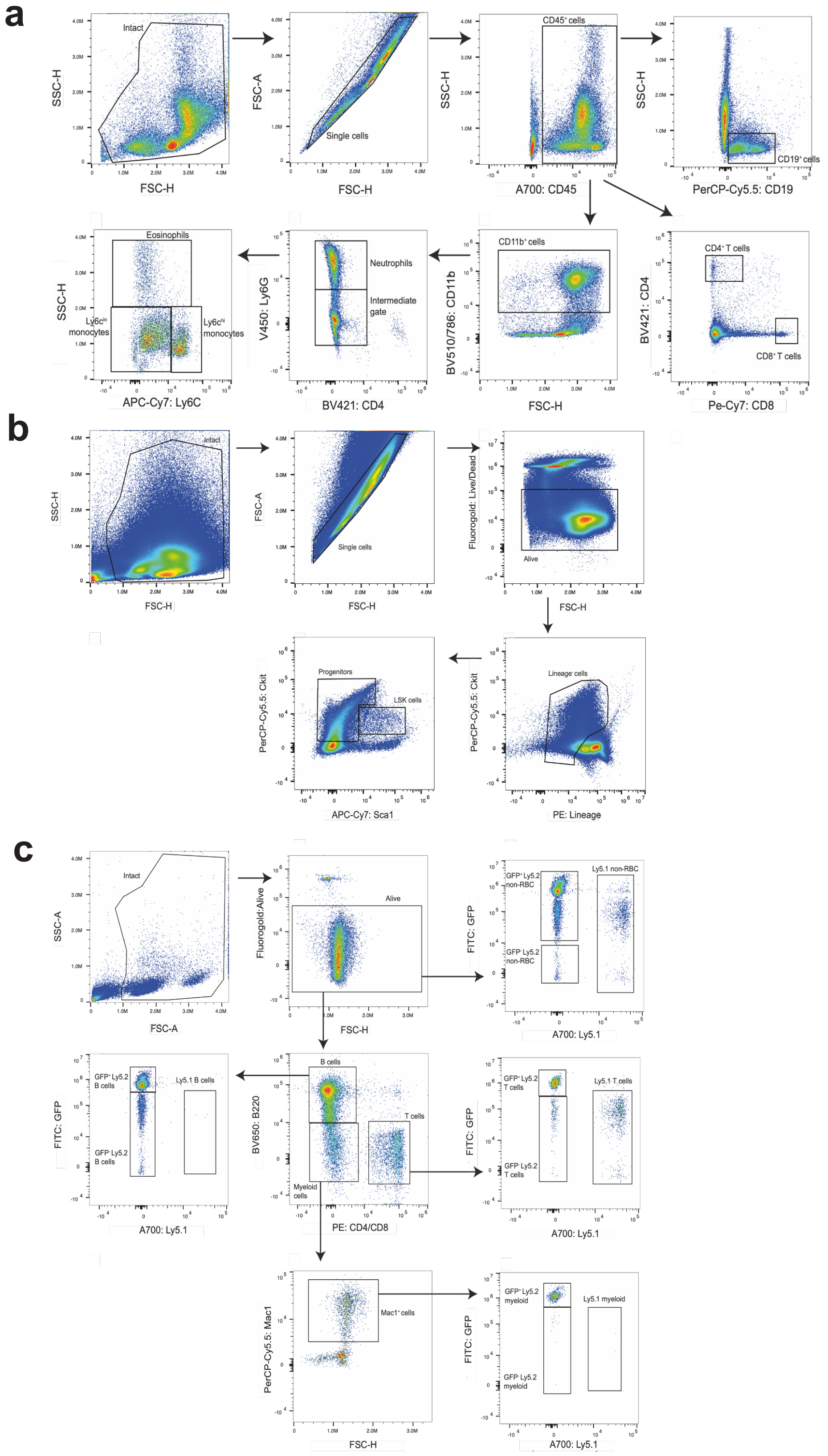

Supplementary Figure 7 (cont)

d

e

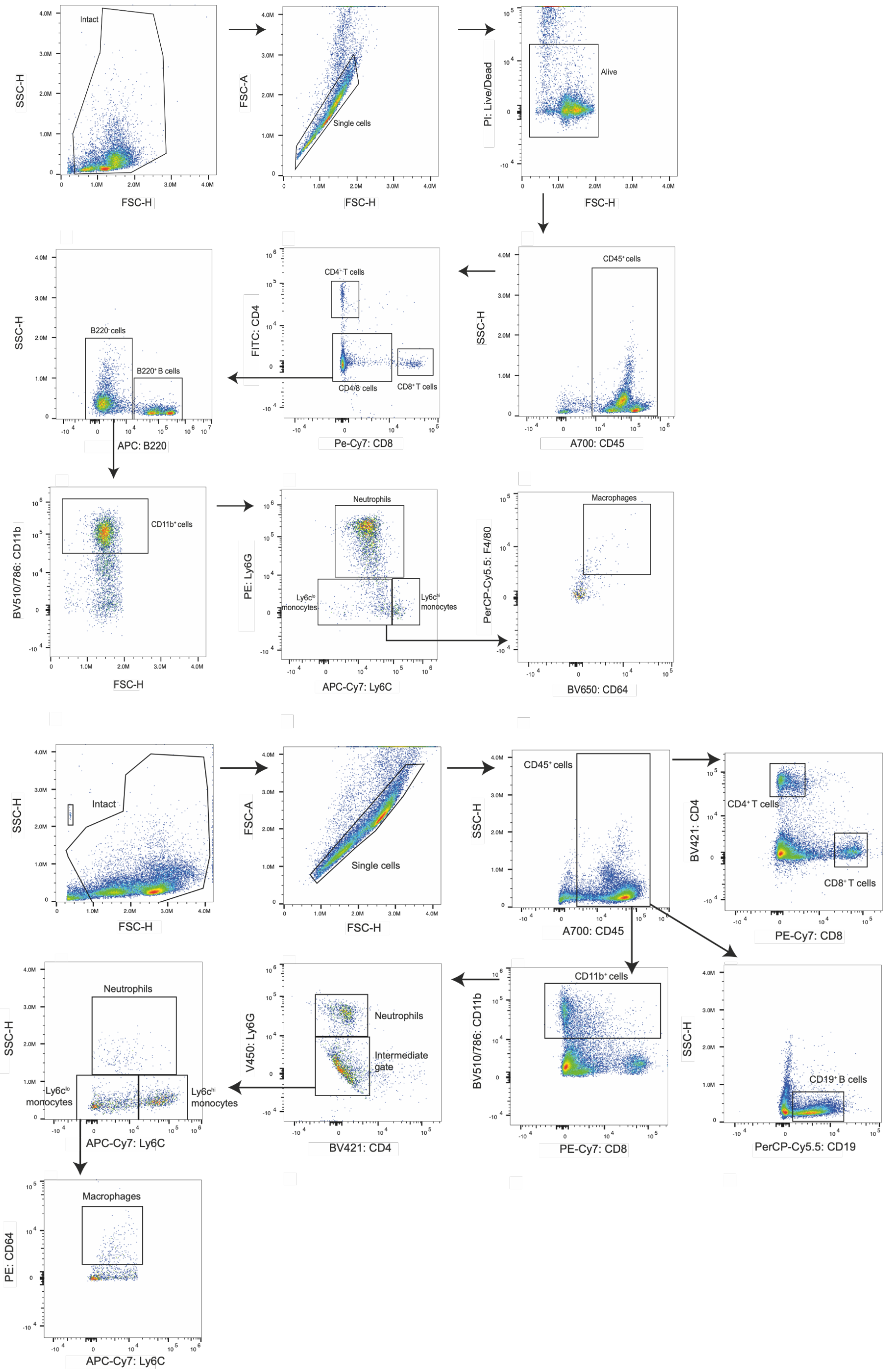

Supplementary Figure 7 (cont)

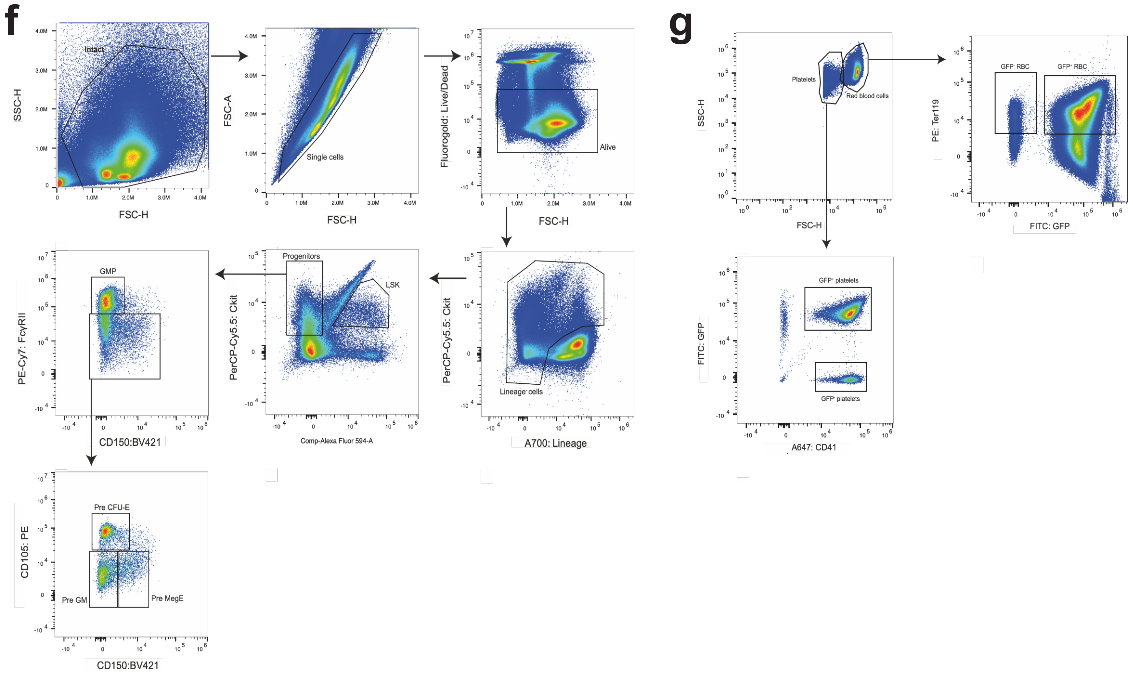

Supplementary Figure 7. Gating strategies used for flow cytometry. Gating strategies correspond to data presented in figures as follows. **a.** Figure 2d-i, Supplementary Figure 2e-s. **b.** Figure 3d-f. **c.** Figure 3h, Supplementary Figure 3h. **d.** Figure 4a-d, Supplementary Figure 4a-p. **e.** Figure 5d, e, Supplementary Figure 5i-o. **f.** Supplementary Figure 2t. **g.** Supplementary Figure 3f, g, i, j.

Supplementary Table 1. List of antibodies used in this study.

Antibody	Source	Identifier	Dilution
Rat anti-mMLKL (8F6)	WEHI	Clone 8F6	1:1000
Rat anti-mMLKL (5A6)	WEHI (Commercially available at Merck-Millipore)	Clone 5A6; MABC1635	1:1000
Rat anti-hMLKL (7G2)	WEHI (Commercially available at Merck-Millipore)	Clone 7G2; MABC1636	1:1000
Rat anti-hRIPK3 (1H2)	WEHI (Commercially available at Merck-Millipore)	Clone 1H2; MABC1640	1:1000
Mouse anti-actin	Sigma-Aldrich	AC15; A-1978	1:5000
Rabbit anti-GAPDH	Cell Signalling Technology	14C10; #2118	1:2000-5000
Rabbit anti-VDAC	Merck-Millipore	AB10527; #2450741	1:10000
Rabbit anti-phospho-hMLKL	Abcam	Ab187091;EPR9514	1:1000-3000
Rabbit anti-phospho--mMLKL	Cell Signalling Technology	D6E3G; #37333	1:1000
Rabbit anti-hRIPK1	Cell Signalling Technology	D94C12; #3493	1:1000
Rabbit anti-GSDMD	Abcam	AB209845; EPR19828	1:1000
CD4-BV421	BD Biosciences	Clone RM4-5; Cat No. 740007	1:200
CD8-PECy7	BD Biosciences	Clone 53-6.7; Cat No. 561097	1:200
CD19-PerCPCy5.5	BD Biosciences	Clone 1D3; Cat No. 551001	1:200
CD11b-BV510	BD Biosciences	Clone M1/70; Cat No. 562950	1:200
CD11b-BV786	BD Biosciences	Clone M1/70; Cat No. 740861	1:200
CD64-PE	BD Biosciences	Clone X54-5/7.1; Cat No.558455	1:400
CD45-Alexa700	BD Biosciences	Clone 30-F11; Cat No.560510	1:400
Ly6G-V450	BD Biosciences	Clone 1A8; Cat No.560603	1:400

Ly6C-APCCy7	BD Biosciences	Clone AL-21; Cat No.560596	1:800
CD41-APC	ThermoFisher	Clone eBioMWReg30; Cat No. 17-0411-82	1:200
Ter119-PE	Conjugated in-house	Clone Ly-76	1:400
Ly5.1-Alexa700	Conjugated in-house	Clone A20.1	1:200
Ly5.2-PE	Conjugated in-house	Clone S450-15-2	1:200
CD4-PE	Conjugated in-house	Clone GK1.5	1:400
CD8-PE	Conjugated in-house	Clone 53-6-7	1:200
B220-BV650	BD Biosciences	Clone RA3-6B2; Cat No. 563893	1:200
Mac1-PerCPCy5.5	BD Biosciences	Clone 104; Cat No. 550993 m1/70	1:200
cKit-PerCPe710	ThermoFisher	Clone 2B8; Cat No. 46-1171-80	1:200
cKit-PerCPCy5.5	BD Biosciences	Clone 2B8; Cat No. 560557	1:400
Sca1-APCCy7	BioLegend	Clone D7; Cat No. 108126	1:400
B220-PE	Conjugated in-house	Clone RA36B2	1:400
CD19-PE	Conjugated in-house	Clone 1D3	1:400
Gr1-PE	Conjugated in-house	Clone RB6-8C5	1:400
Sca1-A594	Conjugated in-house	Clone E13-161.7	1:400
CD150-BV421	Biolegend	Clone TC15-12F12.2; Cat No. 115926	1:200
CD105-PE	ThermoFisher	Clone MJ7/18; Cat No. 12-1051-82	1:200
FcyRII (CD16/32)-PECy7	ThermoFisher	Clone 93; Cat No. 26-0161-82	1:400
CD64-BV650	BD Biosciences	Clone X54-5/7.1; Cat No. 740622	1:300
Ly6G-PE	BD Biosciences	Clone 1A8; Cat No. 551461	1:300
F4/80- PerCPCy5.5	BD Biosciences	Clone T45-2342; Cat No. 567202	1:300
CD4-FITC	BD Biosciences	Clone RM4-5; Cat No. 561835	1:400
B220-APC	BD Biosciences	Clone RA3-6B2; Cat No. 552092	1:200
