# Supplementary material for: A common human MLKL polymorphism confers resistance to negative regulation by phosphorylation
Source: Nat Commun. 2023 Sep 28;14:6046. doi: 10.1038/s41467-023-41724-6 (PMC10539340; doi:10.1038/s41467-023-41724-6)
Supplement: Supplementary file 3 — Reporting Summary [file 41467_2023_41724_MOESM3_ESM.pdf]

nature portfolio

Corresponding author(s): Dr Joanne Hildebrand

Last updated by author(s): Aug 22, 2023

Reporting Summary

Nature Portfolio wishes to improve the reproducibility of the work that we publish. This form provides structure for consistency and transparency in reporting. For further information on Nature Portfolio policies, see our Editorial Policies and the Editorial Policy Checklist.

Statistics

For all statistical analyses, confirm that the following items are present in the figure legend, table legend, main text, or Methods section.

n/a	Confirmed	
☐	☒	The exact sample size (*n*) for each experimental group/condition, given as a discrete number and unit of measurement
☐	☒	A statement on whether measurements were taken from distinct samples or whether the same sample was measured repeatedly
☐	☒	The statistical test(s) used AND whether they are one- or two-sided<br>*Only common tests should be described solely by name; describe more complex techniques in the Methods section.*
☐	☒	A description of all covariates tested
☒	☐	A description of any assumptions or corrections, such as tests of normality and adjustment for multiple comparisons
☐	☒	A full description of the statistical parameters including central tendency (e.g. means) or other basic estimates (e.g. regression coefficient) AND variation (e.g. standard deviation) or associated estimates of uncertainty (e.g. confidence intervals)
☐	☒	For null hypothesis testing, the test statistic (e.g. *F*, *t*, *r*) with confidence intervals, effect sizes, degrees of freedom and *P* value noted<br>*Give P values as exact values whenever suitable.*
☒	☐	For Bayesian analysis, information on the choice of priors and Markov chain Monte Carlo settings
☒	☐	For hierarchical and complex designs, identification of the appropriate level for tests and full reporting of outcomes
☒	☐	Estimates of effect sizes (e.g. Cohen's *d*, Pearson's *r*), indicating how they were calculated

Our web collection on statistics for biologists contains articles on many of the points above.

Software and code

Policy information about availability of computer code

Data collection	Flow Cytometry Aurora Cytex.<br>IncuCyte S3/SX5 Sartorius Imager Software v2022B, v2021B, v2020C & v2018A.<br>CLARIOstar plate reader 5.70 R2
Data analysis	All flow cytometry data were analysed on FlowJo 10.8.1.<br>IncuCyte S3/SX5analysis was completed with Sartorius Imager Software v2022B, v2021B, v2020C & v2018A<br>All data was graphed using Prism v9.

For manuscripts utilizing custom algorithms or software that are central to the research but not yet described in published literature, software must be made available to editors and reviewers. We strongly encourage code deposition in a community repository (e.g. GitHub). See the Nature Portfolio guidelines for submitting code & software for further information.

Data

Policy information about availability of data

All manuscripts must include a data availability statement. This statement should provide the following information, where applicable:

- Accession codes, unique identifiers, or web links for publicly available datasets
- A description of any restrictions on data availability
- For clinical datasets or third party data, please ensure that the statement adheres to our policy

The biological materials generated for MLKL during this study are available from the corresponding authors upon reasonable request. Experimental data generated in this study are provided in the Supplementary Information and Source Data file. Relevant human sequence data can be directly accessed from the Sequence Read Archive (SRA, BioProject accession number PRJNA1007397) via the following link; https://www.ncbi.nlm.nih.gov/sra. Publicly available gnomAD data used can be found at https://gnomad.broadinstitute.org/gene/ENSG00000168404?dataset=gnomad_r2_1. Publicly available PDB data can be found at; 2MSV [https://doi.org/10.1016/j.str.2014.07.014], 4MWI [http://doi.org/10.1042/BJ20131270] and 4BTF [https://doi.org/10.1016/j.immuni.2013.06.018]).

Human research participants

Policy information about studies involving human research participants and Sex and Gender in Research.

Reporting on sex and gender	The study reports on two identified carriers of the p.Ser132Pro polymorphism (patient 1 and 2) and an age- and sex-matched healthy donor control for Patient 2. All three participants were female. No suitable male carriers with the p.Ser132Pro polymorphism were available for this study. Data for only one female participant and their matched health control are presented, and the caveats of such a limited sample size are clearly stated in the discussion section.
Population characteristics	Patient 1 is a female of South American ancestry (self reported), aged in their 30's, and was was diagnosed with SAPHO (synovitis, acne, pustulosis, hyperostosis, osteitis) syndrome. Patient 2 is a female of European ancestry (self reported) aged in their 50's, and was diagnosed with systemic IgG4 disease. Healthy control was age and sex matched to patient #2, but not matched for ancestry. Only data for Patient #2 and healthy age, sex matched control were included in this work
Recruitment	Patients and their relatives were recruited from the Department of Clinical Immunology and Allergy, Royal Melbourne Hospital, Victoria, Australia and the Centre for Personalized Immunology, Australian National University, Canberra, Australia. Unrelated, age and sex matched 'healthy' controls that did not carry the MLKL p.Ser132Pro polymorphism were recruited via the Volunteer Blood Donor Registry, Parkville. Selection of Patients from this registry was based on their genotype, and their geographical location (treated at Royal Melbourne Hospital). No other patient characteristics were considered. Selection of healthy control was similarly based solely on the absence of inflammatory disease, MLKL genotype and their availability to provide a fresh blood sample at the Royal Melbourne Hospital, Melbourne, Australia. There is a potential for self selection bias (capacity to be part of a patient/donor registry and donate blood can reflect level of engagement with healthcare and general fitness levels). These are unlikely to have impacted the results or conclusions reported.
Ethics oversight	All reported procedures were performed with approval from the human ethics review boards of all Institutes that participated in the study; Australian National University, The Walter and Eliza Hall Institute of Medical Research (approved projects 2009.162, 10/02) and in accordance with the 1964 Helsinki declaration and its later amendments or comparable ethical standards.

Note that full information on the approval of the study protocol must also be provided in the manuscript.

Field-specific reporting

Please select the one below that is the best fit for your research. If you are not sure, read the appropriate sections before making your selection.

☒ Life sciences ☐ Behavioural & social sciences ☐ Ecological, evolutionary & environmental sciences

For a reference copy of the document with all sections, see nature.com/documents/nr-reporting-summary-flat.pdf

Life sciences study design

All studies must disclose on these points even when the disclosure is negative.

Sample size	Sample sizes are consistent with field-norms. The numbers of independent repeat experiments are stated in figure legends. Because our prior cell-based studies have shown robust consistency between assays, n of 3 or more were established as sufficient to reveal any differences between cell lines. For animal experiments, as many mice as were available (of appropriate genotype, age and sex) were included. This number varies between experiments but is clearly stated in teach figure legend.
Data exclusions	No data were excluded
Replication	Wherever possible, experimental findings were replicated both through independent experimental repeats (same cell culture used on

	separate days) and through the use of numerous independent biological replicates (cell cultures derived from different mice, performed on the same or different days). Exact n are provided in the legends of all figures.
Randomization	All mice were randomly allocated to experimental groups. Random allocation of experiments not involving animals (cell lines) is consistent with field norms. Cells of each independent line were grown as a single pool until immediately before being counted and assigned to experimental groups randomly.
Blinding	Investigators were blinded to genotype and group allocation during measurement and analyses

Reporting for specific materials, systems and methods

We require information from authors about some types of materials, experimental systems and methods used in many studies. Here, indicate whether each material, system or method listed is relevant to your study. If you are not sure if a list item applies to your research, read the appropriate section before selecting a response.

Materials & experimental systems

n/a	Involved in the study
☐	☒ Antibodies
☐	☒ Eukaryotic cell lines
☒	☐ Palaeontology and archaeology
☐	☒ Animals and other organisms
☒	☐ Clinical data
☒	☐ Dual use research of concern

Methods

n/a	Involved in the study
☒	☐ ChIP-seq
☐	☒ Flow cytometry
☒	☐ MRI-based neuroimaging

Antibodies

Antibodies used

anti-mMLKL, WEHI Clone 8F6
anti-mMLKL, WEHI Clone 5A6 (also commercially available from Merck-Millipore MABC1635) *
anti-hMLKL, WEHI Clone 7G2 (also commercially available from Merck-Millipore MABC1636) *
anti-hRIPK3, WEHI Clone 1H2 (also commercially available from Merck-Millipore MABC1640) *
anti-actin, Sigma-Aldrich A-1978; AC-15
anti-GAPDH, Cell Signalling Technology #2118; 14C10
anti-VDAC, Merck-Millipore, #2450741; AB10527
anti-phospho-hMLKL, Abcam, EPR9514; ab187091*
anti-phospho-mMLKL, Cell Signalling Technology, #37333; D6E3G *
anti-hRIPK1, Cell Signalling Technology, #3493; D94C12*
anti-GSDMD, Abcam, EPR19828; AB209845

Validation

CD4-BV421, BD Biosciences, Clone RM4-5; Cat No.740007- Antibody specifically developed for flow cytometry & commonly used for this application. Validation completed on live mouse splenocytes, and validation information available at https://www.bdbiosciences.com/en-au/products/reagents/flow-cytometry-reagents/research-reagents/single-color-antibodies-ruo/bv421-rat-anti-mouse-cd4.740007
CD8-PECy7, BD Biosciences, Clone 53-6.7; Cat No. 561097- Antibody specifically developed for flow cytometry & commonly used for this application. validation completed on live mouse leucocytes, and validation information available at https://www.bdbiosciences.com/en-au/products/reagents/flow-cytometry-reagents/research-reagents/single-color-antibodies-ruo/pe-cy-7-rat-anti-mouse-cd8a.561097
CD19-PerCPCy5.5,BD Biosciences, Clone 1D3; Cat No. 551001- Antibody specifically developed for flow cytometry & commonly used for this application. Validation completed on live mouse spleen cells, and validation information available at https://www.bdbiosciences.com/en-au/products/reagents/flow-cytometry-reagents/research-reagents/single-color-antibodies-ruo/percp-cy-5-5-rat-anti-mouse-cd19.551001
CD11b-BV510 BD Biosciences Clone M1/70; Cat No. 562950 - Antibody specifically developed for flow cytometry & commonly used for this application. validation completed on mouse bone marrow cells and validation information available at https://www.bdbiosciences.com/en-au/products/reagents/flow-cytometry-reagents/research-reagents/single-color-antibodies-ruo/bv510-rat-anti-cd11b.562950
CD11b-BV786 BD Biosciences Clone M1/70; Cat No. 74086- Antibody specifically developed for flow cytometry & commonly used for this application. Validation completed on mouse bone marrow cells and validation information available at https://www.bdbiosciences.com/en-au/products/reagents/flow-cytometry-reagents/research-reagents/single-color-antibodies-ruo/bv786-rat-anti-cd11b.740861
CD64-PE BD Biosciences Clone X54-5/7.1; Cat No.558455- Antibody specifically developed for flow cytometry & commonly used for this application. Validation completed on mouse bone marrow cells and validation information available at https://www.bdbiosciences.com/en-au/products/reagents/flow-cytometry-reagents/research-reagents/single-color-antibodies-ruo/pe-mouse-anti-mouse-cd64-a-and-b-alloantigens.558455
CD45-Alexa700 BD Biosciences Clone 30-F11; Cat No.560510 - Antibody specifically developed for flow cytometry & commonly used for this application. Validation completed on mouse splenocytes and validation information available at https://

www.bdbiosciences.com/en-au/products/reagents/flow-cytometry-reagents/research-reagents/single-color-antibodies-ruo/alexa-fluor-700-rat-anti-mouse-cd45.560510
Ly6G-V450 BD Biosciences Clone 1A8; Cat No.560603 - Antibody specifically developed for flow cytometry & commonly used for this application. Validation completed on mouse bone marrow cells and validation information available at https://www.bdbiosciences.com/en-au/products/reagents/flow-cytometry-reagents/research-reagents/single-color-antibodies-ruo/v450-rat-anti-mouse-ly-6g.560603
Ly6C-APCCy7 BD Biosciences Clone AL-21; Cat No.560596 - Antibody specifically developed for flow cytometry & commonly used for this application. Validation completed on mouse splenocytes and validation information available at https://www.bdbiosciences.com/en-au/products/reagents/flow-cytometry-reagents/research-reagents/single-color-antibodies-ruo/apc-cy-7-rat-anti-mouse-ly-6c.560596
CD41-APC ThermoFisher Clone eBioMWReg30; Cat No. 17-0411-82- Antibody specifically developed for flow cytometry & commonly used for this application (40 publications). Antibody was verified by cell treatment to ensure the antibody binds to the antigen. Validation completed on mouse platelets and validation information available at https://www.thermofisher.com/antibody/product/CD41a-Antibody-clone-eBioMWReg30-MWReg30-Monoclonal/17-0411-82
B220-BV650 BD Biosciences Clone RA3-6B2; Cat No. 563893 - Antibody specifically developed for flow cytometry & commonly used for this application. Validation completed on mouse splenocytes and validation information available at https://www.bdbiosciences.com/en-au/products/reagents/flow-cytometry-reagents/research-reagents/single-color-antibodies-ruo/bv650-rat-anti-mouse-cd45r-b220.563893
Mac1-PerCPCy5.5 BD Biosciences Clone 104; Cat No. 561114 - Antibody specifically developed for flow cytometry & commonly used for this application. Validation completed on mouse bone marrow myeloid cells and validation information available at https://www.bdbiosciences.com/en-au/products/reagents/flow-cytometry-reagents/research-reagents/single-color-antibodies-ruo/percp-cy-5-5-rat-anti-cd11b.561114
cKit-PerCPe710 ThermoFisher Clone 2B8; Cat No. 46-1171-80 - Antibody specifically developed for flow cytometry & commonly used for this application (40 publications). Validation completed on mouse bone marrow myeloid cells and validation information available at https://www.thermofisher.com/antibody/product/CD117-c-Kit-Antibody-clone-2B8-Monoclonal/46-1171-82
cKit-PerCPCy5.5 BD Biosciences Clone 2B8; Cat No. 560557 -Antibody specifically developed for flow cytometry & commonly used for this application. Validation completed on mouse bone marrow cells and validation information available at athttps://www.bdbiosciences.com/en-au/products/reagents/flow-cytometry-reagents/research-reagents/single-color-antibodies-ruo/percp-cy-5-5-rat-anti-mouse-cd117.560557
Sca1-APCCy7 BD BioLegend Clone D7; Cat No. 108126 -Antibody specifically developed for flow cytometry & commonly used for this application. Validation completed on mouse splenocytes and validation information available at https://www.biolegend.com/en-us/cell-health/apc-cyanine7-anti-mouse-ly-6a-e-sca-1-antibody-6752
CD150-BV421 BioLegend Clone TC15-12F12.2; Cat No. 115926 - Antibody specifically developed for flow cytometry & commonly used for this application. Validation completed on mouse spleenocytes and validation information available at https://www.biolegend.com/fr-fr/products/brilliant-violet-421-anti-mouse-cd150-slam-antibody-7162
CD105-PE ThermoFisher Clone MJ7/18; Cat No. 12-1051-82- Antibody specifically developed for flow cytometry & commonly used for this application (18 publications). Validation completed on bEnd-3 cell line and validation information available at https://www.thermofisher.com/antibody/product/CD105-Endoglin-Antibody-clone-MJ7-18-Monoclonal/12-1051-82
FcyRII (CD16/32)-PECy7 ThermoFisher Clone 93; Cat No. 26-0161-82 – Antibody specifically developed for flow cytometry and commonly used for this application (7 publications). Validation completed on mouse splenocytes and validation information available at https://www.thermofisher.com/antibody/product/CD16-CD32-Antibody-clone-93-Monoclonal/25-0161-82
CD64-BV650 BD Biosciences Clone X54-5/7.1; Cat No. 740622 - Antibody specifically developed for flow cytometry & commonly used for this application. Validation completed in Tan et al., 2003 and validation information available at https://www.bdbiosciences.com/en-au/products/reagents/flow-cytometry-reagents/research-reagents/single-color-antibodies-ruo/bv650-mouse-anti-mouse-cd64-a-and-b-alloantigens.740622
Ly6G-PE BD Biosciences Clone 1A8; Cat No. 551461 - Antibody specifically developed for flow cytometry & commonly used for this application. Validation completed on mouse bone marrow suspension and validation information available at https://www.bdbiosciences.com/en-au/products/reagents/flow-cytometry-reagents/research-reagents/single-color-antibodies-ruo/pe-rat-anti-mouse-ly-6g.551461
F4/80- PerCPCy5.5 BD Biosciences Clone T45-2342; Cat No. 567202 -Antibody specifically developed for flow cytometry & commonly used for this application. Validation completed on mouse splenocytes or mouse peritoneal exudate cells and validation information available at https://www.bdbiosciences.com/en-au/products/reagents/flow-cytometry-reagents/research-reagents/single-color-antibodies-ruo/percp-cy5-5-rat-anti-mouse-f4-80.567202
CD4-FITC BD Biosciences Clone RM4-5; Cat No. 561835 -Antibody specifically developed for flow cytometry, commonly used for this application & routinely teste. Validation information at https://www.bdbiosciences.com/en-au/products/reagents/flow-cytometry-reagents/research-reagents/single-color-antibodies-ruo/fitc-rat-anti-mouse-cd4.561835
B220-APC BD Biosciences Clone RA3-6B2; Cat No. 552092-Antibody specifically developed for flow cytometry & commonly used for this application. Validation completed on mouse splenocytes and validation information available at https://www.bdbiosciences.com/en-au/products/reagents/flow-cytometry-reagents/research-reagents/single-color-antibodies-ruo/apc-rat-anti-mouse-cd45r-b220.553092

Ter119-PE, conjugated in-house by WEHI monoclonal antibody facility (Clone Ly-76).
Ly5.1-Alexa700, conjugated in-house by WEHI monoclonal antibody facility (Clone A20.1).
Ly5.2-PE, conjugated in-house by WEHI monoclonal antibody facility (Clone S450-15-2).
CD4-PE, conjugated in-house by WEHI monoclonal antibody facility (Clone GK1.5).
CD8-PE, conjugated in-house by WEHI monoclonal antibody facility (Clone 53-6-7).
B220-PE, conjugated in-house by WEHI monoclonal antibody facility (Clone RA36B2).
CD19-PE, conjugated in-house by WEHI monoclonal antibody facility (Clone 1D3).
Gr1-PE, conjugated in-house by WEHI monoclonal antibody facility (Clone RB6-8C5).
Sca1-A594 , conjugated in-house by WEHI monoclonal antibody facility (Clone E13-161.7).

All flow cytometry antibodies conjugated in-house by WEHI monoclonal antibody facility were titrated and validated using appropriate target cells (C57Bl/6 splenocytes, bone marrow derived mononuclear cells, thymocytes or Ly5.1 cells). Antibodies were

compared with previous conjugates used and co-stained with unrelated antibodies to check their specificity where possible.

* Validation of antibodies via western blot with respective knockout HT29 cells published in Samson et al., 2021 (DOI: 10.1038/s41418-021-00742-x). Specifically anti-hMLKL 7G2 and anti-phospho-hMLKL EPR9514 validated in Fig 1e, anti-hRIPK3 1H2 validated in Fig 2e, anti-hRIPK1 D94C12 validated in Fig 3E, anti-mMLKL 5A6 and anti-phospho-mMLKL D6E3G validated in Fig 4e.
anti-mMLKL 8F6 produced in house published in Hildebrand et al. 2020 where specificity for mMLKL validated in Fig 1f.

anti-actin, Sigma-Aldrich A-1978; AC-15 - Antibody has been used in publications extensively and validation available at https://www.sigmaaldrich.com/AU/en/product/sigma/a1978, validated for western blot analysis on human foreskin fibroblasts.
anti-GAPDH, Cell Signalling Technology #2118; 14C10 - Antibody has been used in publications extensively and validation is available at https://www.cellsignal.com/products/primary-antibodies/gapdh-14c10-rabbit-mab/2118, validated for western blot analyses on HeLa, NIH/3T3, C6, HUVEC and L929 cell lines.
anti-VDAC, Merck-Millipore, #2450741; AB10527 - Antibody has been used in publications extensively and validation available at https://www.merckmillipore.com/INTERSHOP/web/WFS/Merck-INTL-Site/en_US/-/USD/ShowDocument-File?ProductSKU=MM_NF-AB10527&DocumentId=null&DocumentType=COA&Language=EN&Country=US&ProductBatchNo=2512107&Origin=PDP, validated for western blot analyses on HepG2 cell lysates.
anti-GSDMD, Abcam, EPR19828; AB209845- Validation is available at https://www.abcam.com/products/primary-antibodies/gsdmd-antibody-epr19828-ab209845.html, validated for western blot in RAW 264.7 cells with accompanying knockout cells.

Eukaryotic cell lines

Policy information about cell lines and Sex and Gender in Research

Cell line source(s)	All murine cell lines (MDFs & BMDMs) were derived by the authors themselves from mice generated by the WEHI. Unmodified HT29 cells were supplied by Mark Hampton and originate from ATCC. CRISPR-edited derivative HT29 cells lacking MLKL were reported previously (Petrie et al., Nature Commun 2018) and were produced in-house using unmodified HT29s supplied by Mark Hampton. HEK293T cells were sourced from the laboratory of John Silke, and were originally purchased from the ATCC. MLKL-deficient U937 cells are as described in Petrie et al. Nat Comm 2018 and were produced in-house from parental cells (WT U937) supplied by ATCC. Patient and healthy donor PBMCs were isolated in house as described in the methods from blood donations. BMDMs were derived from a mixture of both male and female mice. Mlkl p.S131P mouse dermal fibroblasts (wt, het and mutant) were derived from pups at E19.5, which can not be accurately sexed using external appearance. Sex chromosomes for these cells lines were not typed.
Authentication	Genotypes of all murine cell lines were identified through genotyping of mice by transnetyx. All murine and human PBMCs were generated in house.<br>None of the HT29 or U937 cells were formally authenticated. However, their morphologies are consistent with the stated cell type. The lack of responsiveness of MLKL-deficient human HT29 and U937 cells to necroptotic stimuli and the lack of respective MLKL reactivity by western blot are consistent with the absence of MLKL.
Mycoplasma contamination	All cell lines used were monitored for mycoplasma and were found to be negative in our routine screening.
Commonly misidentified lines<br>(See ICLAC register)	Nil

Animals and other research organisms

Policy information about studies involving animals; ARRIVE guidelines recommended for reporting animal research, and Sex and Gender in Research

Laboratory animals	MlklS131P CRISPR mouse strain was bred and maintained at WEHI (Parkville) Animal Facility. C57BL/6J mice bred at WEHI Kew facility were used as non-littermate WT controls where indicated. C57BL/6-CD45ly5.1 mice were bred and imported from WEHI Kew facility. Experiments were performed with both male and female MlklS131P colony mice aged from 6 – 52 weeks old, male and female C57Bl/6J mice aged from 6-17 weeks old, and female only C57Bl/6-CD45Ly5.1 mice aged from 6-10 weeks old. Specific age ranges are denoted for each experiment in the methods.
Wild animals	no wild animals were used
Reporting on sex	Both male and female mice were examined for all experiments. Combined sex data is presented in the main figures but all sex separated data is available in supplementary data.
Field-collected samples	no field-collected samples were used
Ethics oversight	The WEHI Animal Ethics Committee approved all experiments in accordance with the NHMRC Australian code for the care and use of animals for scientific purposes.

Note that full information on the approval of the study protocol must also be provided in the manuscript.

Flow Cytometry

Plots

Confirm that:

- [x] The axis labels state the marker and fluorochrome used (e.g. CD4-FITC).
- [x] The axis scales are clearly visible. Include numbers along axes only for bottom left plot of group (a 'group' is an analysis of identical markers).
- [x] All plots are contour plots with outliers or pseudocolor plots.
- [x] A numerical value for number of cells or percentage (with statistics) is provided.

Methodology

Sample preparation	Single cell suspensions from bone marrow, lymph nodes, spleen, and peritoneal lavage were collected from experimental mice as outlined in the methods. Cells were stained with the antibody cocktail outlined in the methods sections. Primary plots are not presented but a numerical value for the number of cell populations is graphed.
Instrument	Aurora Cytex
Software	FlowJo Version 10.8.1<br>Burrows-Wheeler Aligner (BWA-MEM)<br>Genomic Analysis Tool Kit (GATK)<br>IncuCyte SX5 G/O/NIR optical module or S3 G/R optical module<br>BioRad bio-plex data-pro software
Cell population abundance	Data were collected until a regulated number of CD45+ cells were recorded. This number differed between organ examined.
Gating strategy	Gating strategies were devised using guidance from previously published papers including Kauppi M et al. 2008 Blood, Martin et al. 2017 J Immunol. Assistance from established collaborators in the field, Dr Kate Lawlor was obtained verifying validity of gating strategy.<br>All samples were initially gated from "intact" cells using FSC and SSC and for single cells only using FSC-A versus FSC-H. Specific gating for cell populations is outlined in supplementary info.

- [x] Tick this box to confirm that a figure exemplifying the gating strategy is provided in the Supplementary Information.
